# *In vivo *trafficking and immunostimulatory potential of an intranasally-administered primary dendritic cell-based vaccine

**DOI:** 10.1186/1471-2172-11-60

**Published:** 2010-12-10

**Authors:** Prachi Vilekar, Vibhudutta Awasthi, Pallavi Lagisetty, Catherine King, Nathan Shankar, Shanjana Awasthi

**Affiliations:** 1 Department of Pharmaceutical Sciences, University of Oklahoma Health Science Center, 1110 N. Stonewall Avenue, Oklahoma City, OK-73117, USA; 2 Small Animal Imaging Facility, University of Oklahoma Health Science Center, 1110 N. Stonewall Avenue, Oklahoma City, OK-73117, USA

## Abstract

**Background:**

Coccidioidomycosis or Valley fever is caused by a highly virulent fungal pathogen: *Coccidioides posadasii *or *immitis*. Vaccine development against *Coccidioides *is of contemporary interest because a large number of relapses and clinical failures are reported with antifungal agents. An efficient Th1 response engenders protection. Thus, we have focused on developing a dendritic cell (DC)-based vaccine for coccidioidomycosis. In this study, we investigated the immunostimulatory characteristics of an intranasal primary DC-vaccine in BALB/c mouse strain that is most susceptible to coccidioidomycosis. The DCs were transfected nonvirally with *Coccidioides-*Ag2/PRA-cDNA. Expression of DC-markers, Ag2/PRA and cytokines were studied by flow cytometry, dot-immunoblotting and cytometric bead array methods, respectively. The T cell activation was studied by assessing the upregulation of activation markers in a DC-T cell co-culture assay. For trafficking, the DCs were co-transfected with a plasmid DNA encoding HSV1 thymidine kinase (TK) and administered intranasally into syngeneic mice. The trafficking and homing of TK-expressing DCs were monitored with positron emission tomography (PET) using ^18^F-FIAU probe. Based on the PET-probe accumulation in vaccinated mice, selected tissues were studied for antigen-specific response and T cell phenotypes using ELISPOT and flow cytometry, respectively.

**Results:**

We found that the primary DCs transfected with *Coccidioides*-Ag2/PRA-cDNA were of immature immunophenotype, expressed Ag2/PRA and activated naïve T cells. In PET images and subsequent biodistribution, intranasally-administered DCs were found to migrate in blood, lung and thymus; lymphocytes showed generation of T effector memory cell population (T_EM_) and IFN-γ release.

**Conclusions:**

In conclusion, our results demonstrate that the intranasally-administered primary DC vaccine is capable of inducing Ag2/PRA-specific T cell response. Unique approaches utilized in our study represent an attractive and novel means of producing and evaluating an autologous DC-based vaccine.

## Background

Coccidioidomycosis or Valley fever is caused by a dimorphic fungus: *Coccidioides posadasii *or *C. immitis*. Due to high virulence, both of the *Coccidioides *species: *C. posadasii *and *C. immitis *have been included in the National Institute of Allergy and Infectious Disease (NIAID)'s list of Biodefense Pathogens and in the Center for Disease Control (CDC)'s list of Select Agents. Coccidioidomycosis is endemic in areas of Southwest US, Mexico and several countries of South America. The infection is initiated by inhalation of air-borne arthroconidia. An insufficient cell-mediated immunity promotes the formation of parasitic-phase endosporulating spherule structures in lung and hematogenous spread of organisms into non-pulmonary organs leading to more severe disseminated coccidioidomycosis [1]. The disseminated infection causes increased morbidity and mortality, specifically in people with immunocompromised conditions. African-Americans, Fillipinos and pregnant women are also at a high risk of developing disseminated coccidioidomycosis [2]. Among all the endemic fungal infections, coccidioidomycosis has generated a great interest in vaccine development because a prior infection engenders immunity, a large number of relapses and clinical failures are reported with the use of conventional antifungal-agents, the disease produces a significant burden of morbidity, the rate of infection is increasing in endemic areas [3], and most importantly, *Coccidioides *poses a risk of bioterrorism [4].

As is evident from the studies in patients with disseminated coccidioidomycosis and animal models, the susceptibility to the disease is related to defective T cell-immune responses [5]. An effective antigen-presentation by antigen-presenting immune cells is a critical step in engendering protective T cell responses. Among a variety of cells, the dendritic cells (DCs) are the most potent antigen-presenting cells. As such, suppressed DC responses are evidently associated with defective T cell responses in patients with disseminated coccidioidomycosis [6,7], and in susceptible mouse strains, such as BALB/c mouse strain [8,9]. Unlike other antigen-presenting immune cells, DCs migrate to lymph nodes, and activate naïve immune cells including T cells. Based on this property, DC-based vaccines have been evaluated in animal models of a variety of infections as well as cancer. Some of the DC-based vaccines are currently undergoing pre-clinical/clinical trials for AIDS and different types of cancer [10-19]; a therapeutic DC-vaccine (Sipuleucel-T) was recently approved by Food and Drug Administration (FDA) for the management of prostate cancer [20,21]. Our laboratory's focus is on developing a DC-based vaccine for coccidioidomycosis [8,22,23].

The success of a DC-based vaccine depends on multiple factors, including type of antigen, loading efficiency of DCs with antigen, route of administration, trafficking, and the ability to express protective antigen *in vivo*, interact with naïve immune cells and activate effector immune cells. In a previous study, we reported a DC-vaccine prepared by genetically transfecting the immortalized myeloid JAWS II DCs (ATCC, VA) with a plasmid DNA encoding *Coccidioides*-Ag2/PRA-cDNA (a potent protective epitope of *Coccidioides species*) [22]. Furthermore, we showed its protective efficacy as a prophylactic vaccine in reducing the fungal load in syngeneic C57BL6 mouse strain that is moderately susceptible to *C. posadasii *infection [23]. The study provided a proof-of-principle that the non-virally, genetically-transfected DCs can help reduce the fungal load [5]. Based on these initial results [8,22-24], we have now prepared a vaccine using primary myeloid DCs. Besides the possibility of the altered immunostimulatory characteristics of primary DCs in different mouse strain, it is also important to note that an immortalized DC cell line may not be used in clinical scenario. It is also expected that an autologous primary DC-based vaccine will be easily translatable and more feasible as a therapeutic vaccine. Here we used BALB/c mouse strain because it is extremely susceptible to coccidioidomycosis, and *Coccidioides*-infected BALB/c mice present immunological features (less IFN-γ, suppressed DC responses) similar to those observed in patients with disseminated disease [5,8]. Since the efficacy and functions of DC-vaccination depends primarily on DC-phenotypes, it is important to evaluate the phenotype, stability of antigen expression, *in vivo *trafficking, antigen presentation and T cell stimulating potential of primary DCs. With these criteria in mind, a DC-vaccine was prepared using BALB/c mice-derived primary DCs by genetically-transfecting with a plasmid DNA containing *Coccidioides*-Ag2/PRA-cDNA; the phenotype and antigen-presentation were studied. To enable *in vivo *monitoring of DCs by PET imaging, the DCs were co-transfected with HSV1 thymidine kinase cDNA. The image-derived biodisposition of administered DCs assisted in focusing on select organs for further evaluation of memory T cell populations and Ag2/PRA-specific responses.

## Results

### Morphology and Phenotype of primary DCs and JAWS II DCs

**Figure 1:**
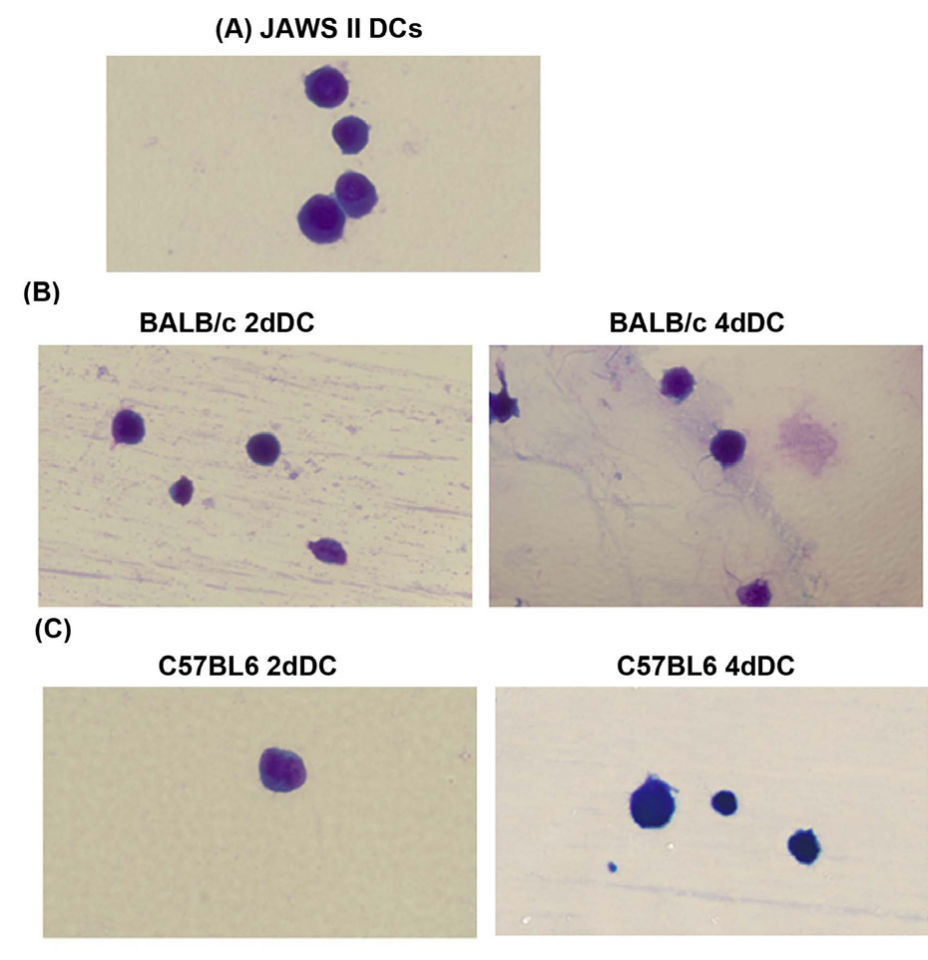
**The morphology of (A) JAWS II DCs, and primary murine DCs derived from (B) BALB/c and (C) C57BL6 mice shows identical features**. The primary DCs were harvested on days 2 (2dDC) and 4 (4dDC) of culturing bone marrow cells. The cells were air-dried on a glass slide, stained with Wright-Giemsa stain, and photomicrographed at 400X using a Leica light microscope.

The yield of primary DCs of BALB/c and C57BL6 mice on day 2 and day 4, varied from 6%-20% of total harvested bone marrow cells. More than 98% of DCs remained viable on day 2 and day 4 of harvesting. Morphologically, 2dDC and 4dDC as well as JAWS II cells showed immature phenotype. There was no difference between the primary DCs originating from either C57BL6 or BALB/c mouse strains. Similar to the JAWS II DCs, the primary DCs did not possess tentacles or dendrite-like structures (Figure 1). When the cells were allowed to continue differentiating for 13 days in presence of GM-CSF and IL-4, the DCs developed typical morphology of mature DCs characterized by the appearance of dendrite structures on their cell surface.

**Figure 2:**
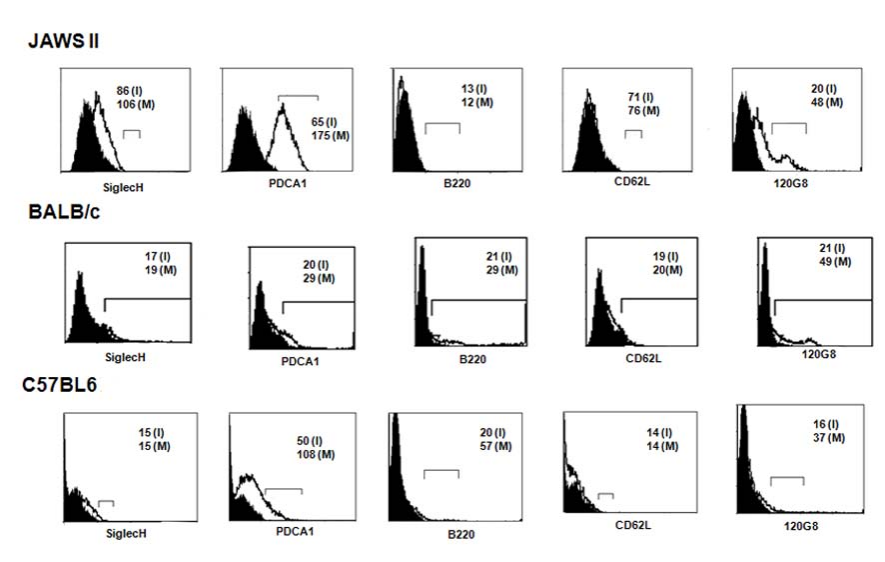
**The flow cytometric histograms of JAWS II DCs and primary murine DCs derived from BALB/c and C57BL6 mice**. The DCs were stained with fluorochrome-conjugated antibodies specific to SiglecH, PDCA1, B220, CD62L, 120G8 antigens. The cell debris was gated-out and histogram charts were plotted. The filled histograms are of isotype-control antibody-stained cells. The mean fluorescent intensity (MFI) values were determined for the cells under the bar (⊓) region. The values within the charts indicate the MFI values for cells stained with isotype-control antibody (I), or antigens-specific antibodies (M). The results are from one representative experiment conducted thrice.

The immunophenotype of harvested primary DCs was confirmed by flow-cytometry. The primary DCs were found to be negative for CD14 (a macrophage marker), CD3 (a T cell marker) and CD45RA (a B cell marker), and this negativity continued through the duration of the experiment. We confirmed the myeloid or plasmacytoid nature of primary DCs by staining with fluorochrome-conjugated antibodies: SiglecH, PDCA1, 120G8, CD62L, B220 and CD11c. As evident from negative or negligible staining for SiglecH, CD62L, B220, the primary DCs and JAWS II DCs were mainly of myeloid phenotype. At the same time, the primary DCs and JAWSII DCs were very low positive for PDCA1 and 120G8 markers demonstrating again that they were not of plasmacytoid type (Figure 2).

**Figure 3:**
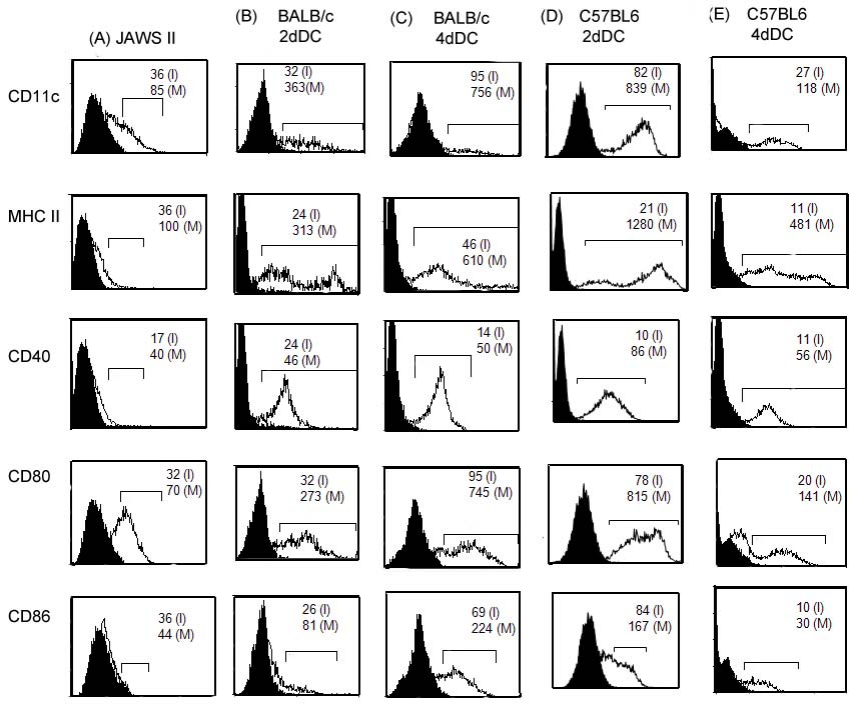
**The flow cytometric histograms of JAWS II DCs and primary murine DCs stained with fluorochrome-conjugated antibodies specific to DC-markers**. The primary DCs were harvested on days 2 (2dDC) and 4 (4dDC) of culture and stained with fluorochrome-conjugated antibodies specific to MHC class II, CD11c, CD40, CD80, CD86 antigens. The cell debris was gated-out and histogram charts were plotted. The filled histogram charts are of cells stained with isotype-control antibody. The mean fluorescent intensity (MFI) values were determined for the cells under the bar (⊓) region. The values within the charts indicate the MFI values for the cells stained with isotype-control antibody (I), or antigens-specific antibodies (M). The results are from one representative experiment conducted thrice.

The JAWS II DCs as well as 2dDC and 4dDC showed positive staining for CD11c, MHC class II and T cell co-stimulatory molecules (CD40, CD80, CD86). However, we observed that compared to JAWS II DCs, the expression of these cell surface markers on primary 2dDCs increased in a time-dependent fashion up to 4 days of monitoring (Figure 3). Based on these observations, we decided to use 2dDCs for downstream experiments.

### Immunophenotype and transgene expression in transfected DCs

**Figure 4:**
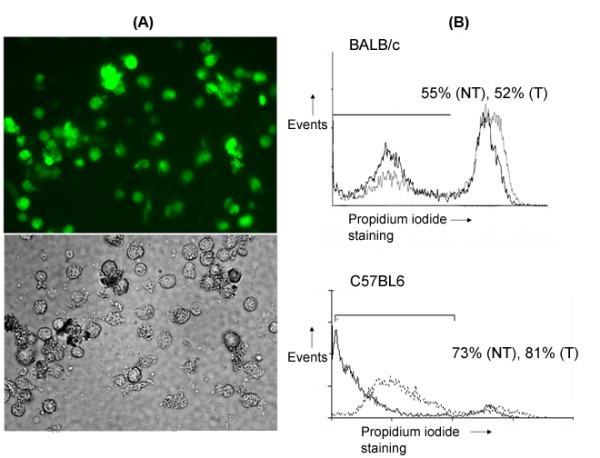
**(A) Photomicrographs of primary DCs after nonviral genetic transfection**. The top panel is a fluorescence photomicrograph of primary DCs showing GFP-expression (in green) after 24 h of transfection. The bottom panel is a photomicrograph of cells in the same field under bright-field filter. (B) Flow cytometric histograms of transfected primary DCs showing the effect of transfection on their viability. The transfected (dotted line) and nontransfected (solid line) primary DCs after two days of culture were stained with propidium iodide. The values indicate % viable nontransfected (NT) and transfected (T) cells under the marked region. The results are from one representative experiment conducted more than three times.

**Figure 5:**
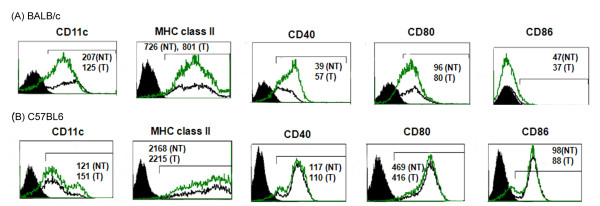
**Immunophenotype of primary DCs derived from (A) BALB/c and (B) C57BL6 mice, does not change after nonviral genetic transfection**. The 2dDC were transfected with pVR1012-Ag2/PRA plasmid DNA. After 24 h of transfection, the cells were stained with fluorochrome-conjugated antibodies specific to MHC class II, CD11c, CD40, CD80, CD86 cell-surface antigens. The cell debris was gated-out and histogram charts were plotted. The filled histogram charts are of cells stained with isotype-control antibody. The mean fluorescent intensity (MFI) values were determined for the cells under the bar (⊓) region. The values within the charts indicate the MFI values for the nontransfected (NT, black line) and transfected (T, green line) cells stained with antibodies specific to cell-surface antigens. The results are from one representative experiment conducted thrice.

Next we investigated whether the primary DCs transfected with plasmid DNAs encoding transgenes expressed the respective proteins, and maintained their immunophenotype. The DNA transfection efficiency of primary DCs ranged from 30-40% which is within 10% of the transfection efficiencies previously reported by us in JAWS II DCs [22] (Figure 4A). The viability of primary DCs after transfection ranged from 52% (versus 55% nontransfected)-81% (versus 73% nontransfected; Figure 4B) as compared to 68% for JAWS II DCs [22]. No significant changes were observed in the cell-surface expression CD11c, MHC class II, CD40, CD80 and CD86 by primary DCs post-transfection (Figure 5). The morphology also remained unaltered (not shown). Similarly, the primary DCs co-transfected with pVR1012-Ag2/PRA-cDNA and pVR1012-TK did not show any changes in cell-surface expression of DC-markers or morphology (data not shown).

**Figure 6:**
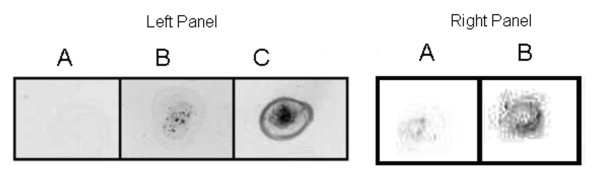
**The expression of transgenes-encoded *Coccidioides*-Ag2/PRA and TK antigens by genetically-transfected primary DCs**. The cell-lysates were prepared after 24 h of transfection, and dot-immunoblotting was performed with Ag2/PRA and TK-specific antibodies. **Left Panel**: An Ag2/PRA dot-immunoblot of 5 μg cell-lysate protein from nontransfected (A), transfected primary DCs (B), and 0.2 μg recombinant Ag2/PRA protein (C). **Right panel**: A TK Dot-immunoblot of 1 μg cell-lysate protein from nontransfected (A) and transfected DCs (B). Results are from one experiment representative of more than three experiments.

We confirmed the expression of Ag2/PRA and HSV1-TK proteins by dot-immunoblotting of cell-lysate of primary DCs co-transfected with pVR1012-Ag2/PRA-cDNA and pVR1012-TK (Figure 6).

### Cytokine secretion by transfected DCs

**Figure 7:**
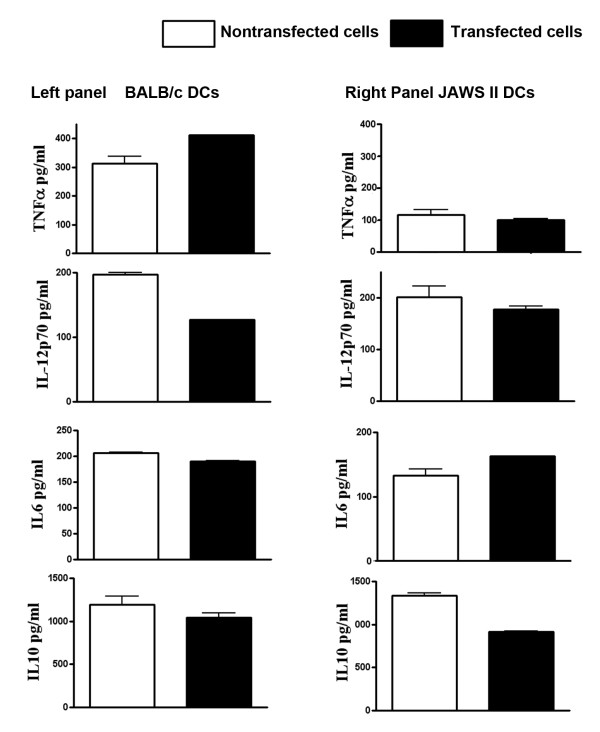
**Cytokine (TNF-α, IL-12p70, IL-6 and IL10) levels (pg/ml) in cell-free supernatants of JAWS II and BALB/c mice derived primary DCs after 24 h of genetic transfection with pVR1012-Ag2/PRA plasmid DNA**. Results are from two experiments performed in triplicate.

After characterizing the transfected primary DCs for their phenotype, we measured the levels of various cytokines in cell-free supernatants. No significant changes were observed in secreted amounts of TNF-α, IL-6, IL-12p70 and IL-10 (Figure 7).

### Primary DCs transfected with pVR1012-Ag2/PRA-cDNA induce activation of both CD4+ and CD8+ T lymphocytes

**Figure 8:**
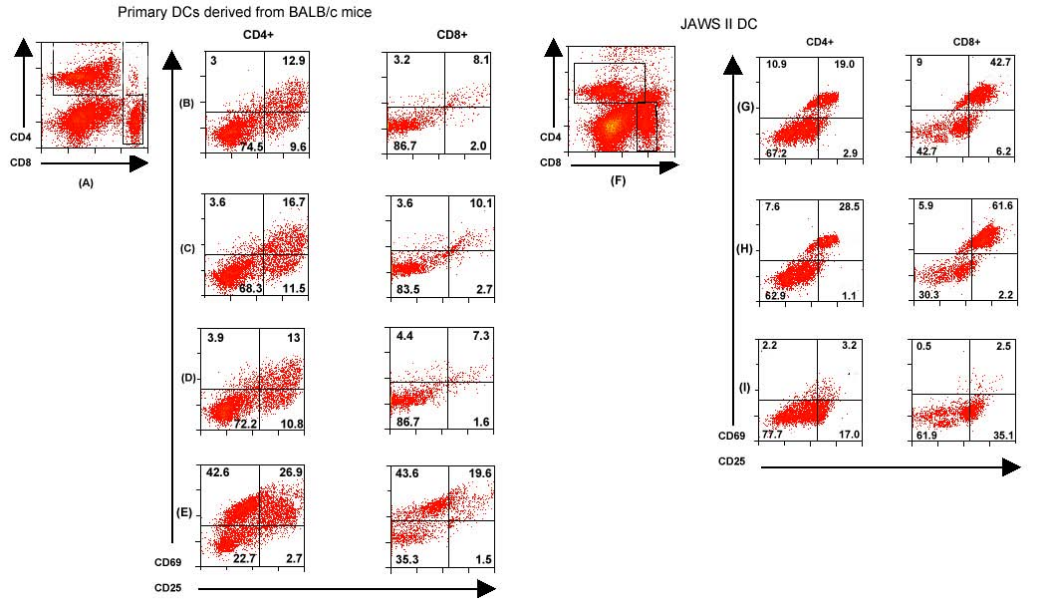
***In vitro *T cell activation by *Coccidioides*-Ag2/PRA-cDNA transfected DCs**. Panels (A) to (E) are BALB/c mice derived primary DCs, and panels (F) to (I) are JAWS II DCs. The vector-plasmid DNA-transfected DCs (panels B and G) and *Coccidioides*-Ag2/PRA-cDNA transfected DCs (panels C and H) were co-cultured with autologous splenic lymphocytes (1DC: 16 lymphocytes) for 24 h. The co-cultured cells were stained with CD4, CD8, CD25 and CD69-specific antibodies. Panels (A) and (F) are dot plots for CD4+ and CD8+ cells. Representative dot-plots of splenic lymphocytes (without DCs, as negative control) are shown in panels (D) and (I). Positive control included concavalin-treated splenic lymphocytes (panel E). The values in each quadrant represent the percent number of CD4+ and CD8+ cells. Results are from one experiment representative of three experiments performed in duplicate.

The primary goal of DC-vaccines is to stimulate lymphocyte-mediated immunity. Therefore, we investigated whether the transfected primary DCs are capable of activating homologous lymphocytes *in vitro*. We harvested cells after 24 h of DC-lymphocyte co-culture for flow cytometric analysis. The cells were stained with fluorochrome-conjugated antibodies against CD4, CD8 (T cell markers), CD25 and CD69 (T cell activation-specific markers) and analyzed by 4-color flow cytometry. Our results suggest that the BALB/c-derived primary DCs induce activation of both CD4+ and CD8+ T cells (Figure 8). The induction of CD4+ and CD8+ T cells was more pronounced with JAWS II DCs as compared to BALB/c mice-derived primary DCs. The concavalin-A activated splenic lymphocytes, DCs and lymphocytes alone served as controls in these experiments (Figure 8).

### Molecular imaging of primary DCs in a mouse model

After comparing the morphology, immunophenotype, expression of the epitope, cytokine release and T cell-stimulatory characteristics of pVR1012-Ag2/PRA-cDNA-transfected primary DCs, we extended our study to investigate the trafficking of primary DCs. We used an HSV1-TK/^18^F-FIAU system to image the distribution of transfected primary DCs in a syngeneic mouse model.

#### Uptake of FIAU by JAWS II DCs transfected with pVR1012-TK

**Figure 9:**
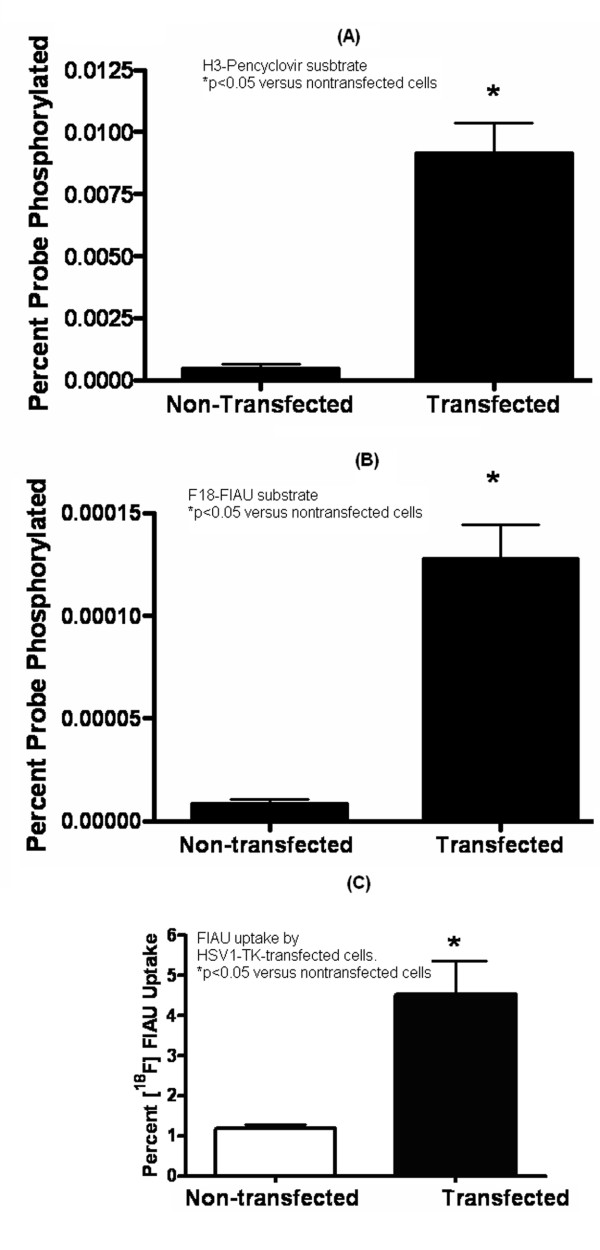
***In vitro *thymidine kinase (TK) enzyme activity in JAWS II DC transfected with pVR1012-TK**. The enzyme activity was measured in the cell lysates using (A) ^3^H-pencycloviir, a conventional substrate for HSV1-TK enzyme, and (B) ^18^F-FIAU. The enzyme expression was confirmed by studying the (C) uptake and retention of ^18^F-FIAU in the transfected cells after 4 h of adding the radiolabeled substrate. * p < 0.05 versus nontransfected cells. Results shown here are mean (± SEM) of three experiments.

First, we determined the HSV1-TK enzyme activity in DC cell lysates based on their ability to phosphorylate ^3^H-Pencyclovir and ^18^F-FIAU substrates. We found that the HSV1-TK-expressing primacy DCs phosphorylate these substrates, and the phosphorylated substrate retained on the filter (Figure 9A-B). In cellular uptake studies, we found that HSV1-TK-transfected JAWS II DCs accumulated 3.8 folds more ^18^F-FIAU (Figure 9C). Overall, the results suggest that HSV1-TK-transfected DCs express an active TK enzyme that is capable of phosphorylating and retaining ^18^F-FIAU substrate.

#### *In vivo *trafficking of primary DCs

**Figure 10:**
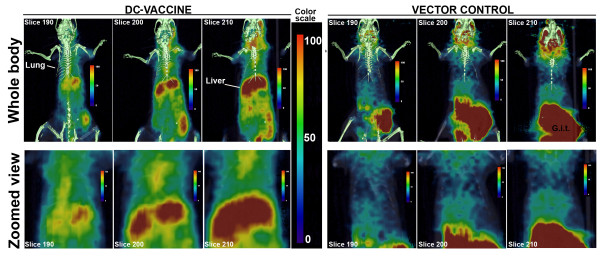
**Fused positron emission tomography (PET) and computed tomography (CT) images of BALB/c mice intranasally instilled with homologous primary DCs expressing HSV1-TK**. The primary DCs were either co-transfected with pVR1012-Ag2/PRA-cDNA and pVR1012-TK (labeled as DC-vaccine), or with vector plasmid DNA (labeled as Vector Control). The PET-CT imaging was performed after 7 days of cells administration. About 2 h prior to imaging, ^18^F-FIAU (a substrate for HSV1-TK) was intravenously injected. Images are from one representative mouse from each group. Six mice were included per group in three different experiments. In the second panels of each group, the whole body images were zoomed to get closer view of ^18^F-FIAU accumulation (DC-migration) in thoracic area. A significant accumulation of ^18^F-FIAU was observed in lung and liver of mouse injected with DCs expressing TK. Overall, the mice receiving DC-vaccine retained more ^18^F-FIAU radioactivity than the Control mice. G.i.t (gastrointestinal tract).

For a successful DC-vaccine, it is important that the cells traffic through or home into the areas where they can present the antigens to naïve immune cells. Also, for an adequate immune response the persistence of the DCs in the body for a reasonable time period is also essential. Based on our previous published results [5], we intranasally instilled about 1.5 million transfected primary DCs in syngeneic mice. The instilled DCs were previously co-transfected with *Coccidioides*-Ag2/PRA-cDNA and pVR1012-TK. For the sake of simplicity, we will call the group of mice instilled with *Coccidioides*-Ag2/PRA-cDNA+pVR1012-TK plasmid transfected DCs as 'DC-vaccine group'; similarly, the mice that received DCs transfected with vector plasmid DNA are called 'Control'. We studied the homing of DCs in live mice using PET-CT imaging after 2 and 7 days of DC administration. Co-registration of CT assisted in ascertaining anatomical landmarks on the fused images (Figure 10). In both 'DC-vaccine' as well as 'Control' groups on day 2, the images did not show significant accumulation of radioactive probe in the mice bodies. However, the PET images acquired on 7^th ^day showed a significant accumulation of ^18^F-FIAU in lung and liver of 'DC-vaccine' as compared to 'Control group' (Figure 10).

#### Biodistribution

**Figure 11:**
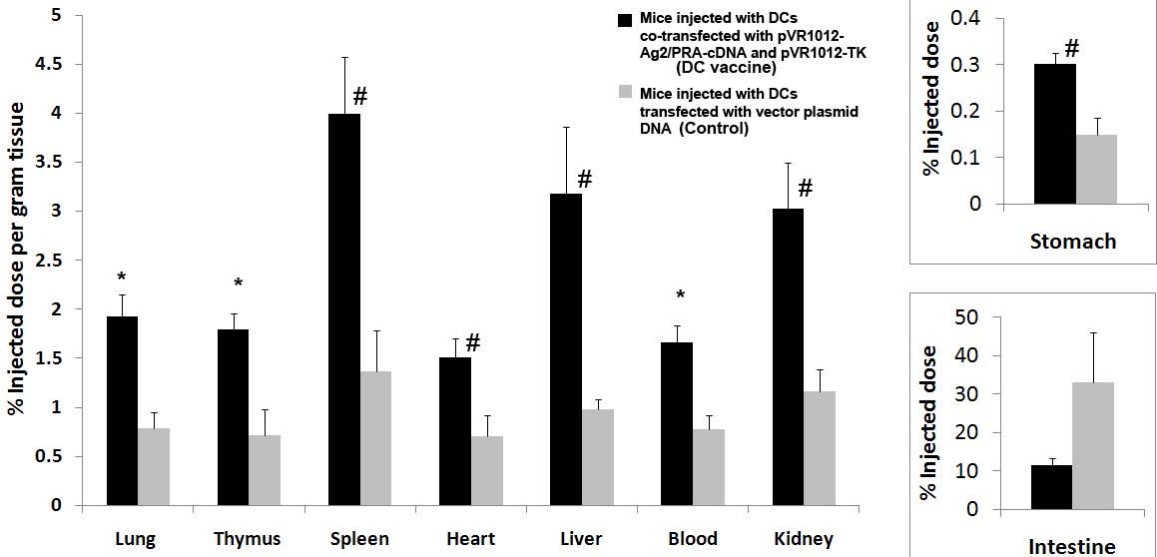
**The biodistribution of ^18^F-FIAU radioactive counts in major organs and fluid specimens of mice**. The animal model and the treatment are described in the figure 10 legend. Mice were euthanized after 140 min of injecting ^18^F-FIAU probe, and major organs and fluids were harvested. The radioactive counts were corrected for physical decay of ^18^F (half-life 110 min), and normalized with tissue weight. The intestine and stomach (insets) were calculated as percent injected dose for entire organ, because of the unavoidable problem related to the presence of variable amounts of food and fecal matter significantly altering the tissue weight from one animal to the other. * p < 0.005, # p < 0.05 versus control (mice injected with DCs transfected with vector plasmid DNA). Results shown here are mean (SEM) of three experiments with 6 mice in each group.

On day 7 after the imaging was accomplished, the mice were euthanized to collect various organs and fluid specimens. The organs were weighed and ^18^F-radioactivity was counted. The biodistribution data confirmed the PET-CT imaging results. There was significantly more radioactivity in all the organs harvested from 'DC-vaccine' group as compared to the 'Control' group. Due to the difficulty in normalizing intestine- and stomach-associated radioactivity by weight, we report the amount of radioactivity in entire organ for these two organs. While intestine did not show significant difference between the two groups, stomach of 'DC-vaccine' group accumulated higher amounts of radioactive probe than the 'Control' group. The differences were more pronounced in lung, thymus and blood (p < 0.005) in the two groups (Figure 11).

### Memory T cell phenotypes and Ag2/PRA-specific response

After assessing the trafficking of the primary DC-vaccine, the T cell phenotypes were enumerated in lymphoid organs: spleen, thymus and lymph nodes. We found that there was a slight decrease or no change in number of CD4+ and CD8+ T_CM _and resting memory cell populations, respectively, with simultaneous increase in T_EM _populations in splenic lymphocytes and lymph node cells of vaccinated mice (Table 1). However, no significant difference was observed in the number of activated (CD25+ CD69+/-) cells.

**Table 1:** Phenotype of splenic lymphocytes, thymocytes and lymph node cells (as percent CD4+ and CD8+ gated) harvested from DC-vaccinated and control mice after 7 days of first immunization.

Cells→ Groups ↓	CD4+				CD8+			
	
	CD25+ CD69+/- activated T cells	CD44+ resting memory	T_EM_ CCR- CD62L hi(lo)	T_CM_ CCR+ CD62L hi	CD25+ CD69+/- activated T cells	CD44+ resting memory	T_EM_ CCR- CD62L lo	T_CM_ CCR+ CD62L hi
**Spleen**								
DC vaccinated	0.88	87.3	**39.9(56)**	**3.5**	99.9	88.5	**27.4**	**6.8**
Control	1.27	92.6	**56(38.2)**	**6.0**	100	83.3	**20.1**	**9.8**

**Thymocytes**								
DC vaccinated	15.9	85.7	27.4(71)	0.51	100	53.8	69.3	3.5
Control	18.9	86.1	26.4(72)	0.53	100	56.8	69	3.7

**Lymph node**								
DC vaccinated	1.72	88	**64(33)**	**2.3**	100	72.2	**34.2**	**3.2**
Control	1.51	90.2	**67(29)**	**3.2**	100	74.4	**32**	**3.9**

Results are from one representative experiment (n = 5 mice per group) performed thrice.

**Figure 12:**
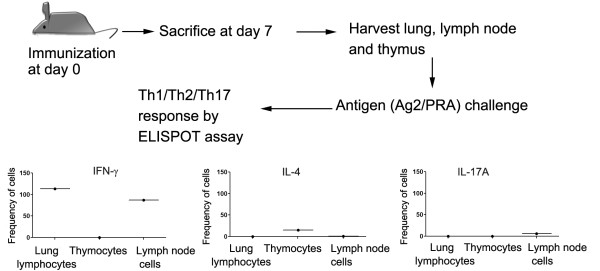
**Assessment of Ag2/PRA-specific response in DC-vaccinated mice**. The antigen-recall response was assessed by ELISPOT assay in lymphocytes harvested from lung, thymus and lymph nodes of DC-vaccinated mice. The numbers of IFN-γ, IL-4 and IL-17A-secreting lymphocytes were counted by ELISPOT assay. The spots obtained were normalized per one million cells and subtracted those from control (mice injected with DCs transfected with vector plasmid DNA). The results shown here are mean (SEM) values obtained from 5 mice in each group.

Finally, we assessed the Ag2/PRA-specific T cell response in lung, thymus and lymph node cells harvested from vaccinated mice. We found that a significant number of lung and lymph node cells derived from vaccinated mice secreted IFN-γ in response to Ag2/PRA challenge. However, there was no significant difference in numbers of IL-4 and IL-17A-secreting cells (Figure 12).

## Discussion

Here, we present our recent data on primary bone-marrow-derived DCs genetically-transfected with *Coccidioides*-Ag2/PRA in BALB/c mouse strain that is most susceptible to *Coccidioides *infection. Compared to our earlier study on immortalized JAWS II DCs [23], the transfection efficiency, viability and immunophenotype of primacy DCs was essentially identical. The transfected primary DCs expressed almost similar levels of GFP, Ag2/PRA and TK protein for at least up to 72 h of transfection under *in vitro *conditions (Figures 4 and 6). These results confirmed that a non-viral method is equally efficacious for genetic transfection of primary DCs as had been observed for immortalized JAWS II cells [22,23]. Empirically, the generation of a protective immune response by a DC-based vaccine depends mainly on its phenotype and antigen-presenting functions that may differ among the mouse strains [13,25-28]. Therefore, we studied basic phenotypic characteristics, antigen-presentation, *in vivo *trafficking of primary DC-based vaccine in BALB/c mouse strain and the ability of primary DC-based vaccine to induce antigen-specific T cell response.

Our results suggest that primary DCs are morphologically similar to JAWS II DCs under resting conditions (non-transfected). At phenotypic level, we found that the primary DCs were mainly of myeloid DC type, like JAWS II DCs, but there were slight differences in the expression pattern of certain cell-surface markers. For instance, the expression of CD11c, MHC class II and CD80 (all myeloid-specific markers) was more pronounced on the cell-surface of primary DCs as compared to JAWS II DCs (Figure 3). The difference in expression pattern of myeloid DC-specific markers may be due to the differences in culture conditions. As noted above, JAWS II DCs were maintained in culture medium containing GM-CSF only, whereas the primary DCs were cultured in the presence of GM-CSF as well as IL-4. Earlier, Jiang et al., found similar differences in the expression of CD11c, MHC II and CD80 between resting JAWS II DCs and C57BL6 mice-derived primary DCs [29].

Under *in vitro *culture-conditions, the DCs mature over a period of time [8,30]. Thus, the timing of DC culture and harvest needs optimization on case-by-case basis. Since we did not observe any significant difference between the antigen presenting ability of the 2dDC and the 4dDC, we used 2dDC for genetic transfection and immunization. We also observed that CD11c, MHC class II and T cell co-stimulatory molecules (CD40, CD80 and CD86) continued to increase in primary DCs from day 2 to 4. Based on these comparisons of phenotypic and functional analysis, we chose 2dDCs for antigen-presentation and downstream *in vivo *immunization experiments. We believe that it may ultimately be beneficial in clinical scenarios to obtain the starting material, i.e., primary DCs within 2 days of seeding the bone marrow cells in DC-promoting culture conditions.

We further studied the functional activity of primary DC-vaccine by DC: T cell co-culture assay. The activation of autologous CD4+ and CD8+ T cells was evident in co-culture assays (Figure 8). However, the expression of CD25 and CD69 was more pronounced in CD4+ and CD8+ T cells co-cultured with C57BL6-derived JAWS II vaccine as compared to BALB/c-derived DC- vaccine. We were intrigued by this finding and decided to explore the cytokine secretion. We observed no significant difference in cytokine (TNF-α, IL-6, IL-10, IL-12) secretion by pVR1012-Ag2/PRA-cDNA transfected primary DCs as compared to similarly transfected JAWS II DCs. It is however, apt to mention that we, and others, have found significant differences in DC-responses against *Coccidioides *in different mouse strains, specifically BALB/c versus DBA/2 and C57BL6 versus DBA/2 [8,9]. Differences in immune responses elicited by different immunization strategies against *Mycobacterium tuberculosis *[31] and *Porphyromonas gingivalis *[32,33] have also been reported in C57BL6 and BALB/c mouse strains. It appears that host genetic factors may be responsible for the differences in antigen-recognition and immune responses in the two mouse strains.

The entire *in vitro *work discussed thus far pointed towards an effective primary DC vaccine. The first step to realizing this *in vivo *is to study the distribution and homing of DCs in appropriate tissues and activation of antigen-specific immune response. To accomplish their biological functions, the DCs undergo a complex pattern of migration which includes their localization to both peripheral non-lymphoid tissues and secondary lymphoid organs. In the absence of correct tissue localization, the DCs fail to promote proper immune responses [34-37]. Thus, we studied the trafficking and homing pattern of primary DC-vaccine in BALB/c mouse strain. The short-term trafficking aspect has already been addressed for a C57BL6-derived JAWS II vaccine in a syngeneic C57BL6 mouse model in our earlier study [23]. In our published study, we labeled the JAWS II vaccine with ^111^In radionuclide and followed the trafficking of cells for a period of 72 h using SPECT (Single photon emission computer tomography). However, we noted some technical limitations with ^111^In-SPECT for DC-trafficking. One, it does not ensure the integrity of radiolabel and DC association *in vivo*, second the resolution is poor, and lastly it allows imaging only up to 3-4 days. To overcome these limitations, here we used a molecular PET imaging approach. This is the first time we have been able to study DC-trafficking *in vivo *up to 7 days of administration. Similar approach however, has been used for imaging the migration of other immune cells and stem cells over a period of 28 days [38-41]. The PET-CT images and the subsequent biodistribution studies suggested that after intranasal administration, significant number of DCs accumulate in lung, thymus and blood. Although the life-span of endogenous DCs is believed to be short, it is not exactly known how long the DCs survive *in vivo *after administration [42-44]. Our results suggest a likelihood that the primary DC-based vaccine can circulate in the body for at least 7 days of immunization.

Finally, we questioned if the homing of intranasally-administered DC-based vaccine in lung and lymphoid organs is sufficient to induce antigen-specific T cell response and memory. Sufficient evidence exists to support the fact that generation of immunological memory is important for a long-term protection [45,46]. Using a multi-color flow-cytomteric approach, we found a consistent increase in the number of CD4+ and CD8+ T_EM _cell population in vaccinated mice suggesting that the DC-vaccine induces an immunological memory. Since the conversion of naïve T cells to memory cells is a dynamic process and involves multiple steps, our efforts will be to investigate the time-dependent analysis of memory T cell distribution. The increased secretion of IFN-γ by lung and lymph node cells correlates well with our previously published results on increased levels of IFN-γ in lung tissue homogenates of DC-vaccinated, *Coccidioides*-protected mice [23]. Our findings may have direct clinical relevance because the reduced levels of IFN-γ cytokine and T cell anergy are associated with disseminated coccidioidomycosis in human patients and animal models [5,47].

## Conclusions

Overall, our results suggest that the primary DC-vaccine can be prepared by using a simple method of nonviral genetic-transfection, first developed in our laboratory [22,23]. After intranasal administration, the DC-vaccine migrates to both lymphoid and non-lymphoid organs, induces antigen-specific Th1 response, and generates memory T cells. Efforts are underway to further evaluate maintenance of immune responses and memory on long-term basis and efficacy of DC-vaccine in *Coccidioides *infection model in BALB/c mouse strain. As described earlier, the *Coccidioides*-specific DC responses and resulting T cell functions are disabled in the BALB/c mouse strain making them highly susceptible to *Coccidioides *infection [8]; the adoptive transfer of DC-vaccine may restore the immunocompetence and contribute to a protective T cell response in infection model.

## Methods

### Mice

We used six weeks old female BALB/c and C57BL6 mice (Jackson Laboratories, ME). All procedures, involving animals, were approved by the Institutional Animal Care and Use Committee of the University of Oklahoma Health Science Center. An acclimatization period of one week was allowed to the animals prior to any experiment.

### Culture of murine bone marrow-derived primary DCs and JAWS II DCs

Primary DCs were obtained by culturing the murine bone marrow cells that were harvested as per the method described earlier [8,48]. The harvested bone marrow cells were cultured in RPMI 1640 medium (Gibco Life Sciences, NY) containing 10 mM N-2-Hydroxyethylpiperazine-N'-2-ethanesulfonic acid (HEPES), 10 μg/ml gentamicin, 100 U/ml penicillin, 100 μg/ml streptomycin, 10% fetal bovine serum (FBS), 1% MEM nonessential amino acids, 50 μM β-mercaptoethanol, 10 ng/ml recombinant mouse-GM-CSF and 10 ng/ml recombinant mouse-IL-4 (both cytokines from Peprotech, NJ). The cells were incubated at 37°C in 5% CO_2 _atmosphere and the DCs were harvested either on day 2 or day 4 on Optiprep density gradient solution (Accurate Chemicals, NY) [30]. Briefly, nonadherent cells were collected, washed and subjected to density gradient separation. The DCs were isolated as floating cells from the top layer (density < 1.065 g/ml) of the Optiprep density gradient.

JAWS II cells are an immortalized immature myeloid DC cell line derived from the bone marrow of p53-/- C57BL6 mice. We obtained JAWS II cells from ATCC, VA, and maintained them in complete Alpha MEM medium containing 20% FBS, 5 ng/ml GM-CSF and antibiotics [22,23].

### Isolation of splenic lymphocytes

The lymphocytes were isolated from murine spleen using Lympholyte-M solution (Cedarlane, Canada) as per the manufacturer's instructions. Briefly, spleen was minced between two sterile glass slides, and a single cell suspension was obtained by passing the minced spleen through a nylon filter (BD Biosciences, CA). Five ml of the splenic cell suspension (~1 × 10^7 ^cell/ml in Hanks balanced salt solution) was layered over 5 ml of Lympholyte M and centrifuged for 20 min at 1000-1500 × g. After centrifugation, the lymphocytes were collected from the interface and washed twice, prior to further analysis.

The viability of cells was determined by standard trypan blue dye exclusion test. The cell morphology was observed by staining the air-dried cells with Diff-Quik stain (Dade Behring, IL).

### Preparation of Plasmid DNA clones

A plasmid DNA clone encoding full-length *Coccidioides*-Ag2/PRA-cDNA (pVR1012-Ag2/PRA) was used. We also included pHYG-EGFP (BD Biosciences Clontech, Palo Alto, CA) for measuring the transfection efficiency. In addition, we prepared a plasmid pVR1012-TK clone that carried HSV-1 encoded thymidine kinase (TK). The HSV-1-TK insert was amplified from the pMOD-TK (Invivogen, CA; with low CpG content) using forward primer 5'AAAACTCGAGTCACTATAGGAGGGCCACCA3' carrying the PstI restriction site and reverse primer 5'AGCAAAAAAAGCTCAGCA3' carrying the BamHI restriction site. The PCR amplified HSV-1 TK insert was digested with BamHI and PstI restriction enzymes and subcloned into the pVR1012 vector plasmid DNA (Vical, CA) to obtain pVR1012-TK. The subcloning of the TK insert (1149 bp) was confirmed by restriction digestion. The in-frame cloning was confirmed by sequencing the pVR1012-TK plasmid DNA clone at the Sequencing facility (Oklahoma Medical Research Foundation, Oklahoma City, OK). The endotoxin-free plasmid DNAs were prepared using Endo-free Maxiprep kit (Qiagen, CA).

### Transfection of primary DCs and JAWS II DCs

The primary DCs (2dDC harvested on day 2 and 4dDC harvested on day 4 of culture) and JAWS II DCs were washed with serum free Dulbecco's minimum essential medium (DMEM; Invitrogen, CA) before transfection. A non-viral, lipid-based transfection reagent: TransIT-TKO (Mirus Bio, WI) was used. The transfection reagent-DNA complex (4 μl:2 μg of each plasmid DNA) was prepared in DMEM medium by adding plasmid DNAs (2 μg pHYG-EGFP or 2 μg pVR1012-VP22 vector plasmid DNA or 2 μg pVR1012-Ag2/PRA-cDNA ± 2 μg pVR1012-TK) to the TransIT-TKO reagent. The cells were incubated with the lipid/DNA mixture at 37°C in 5% CO_2 _incubator. The percent transfection efficiency was evaluated by visual enumeration of the green fluorescent transfected cells versus total number of cells using a fluorescent microscope with appropriate filter (Olympus Optical Co. Ltd, Tokyo, Japan). The viability of transfected cells was assessed by flow cytometry after staining the cells with propidium iodide (1 μg/ml).

The protein expression of Ag2/PRA and HSV-1 TK in co-transfected cells was studied by dot-immunoblotting with Ag2/PRA- and TK-specific antibody (Santacruz Biotech, CA), respectively [22]. The recombinant Ag2/PRA protein served as a control. The recombinant Ag2/PRA-protein and specific antibody were obtained from Dr. John Galgiani (University of Arizona, Tucson, AZ). The HSV-1 TK antibody (Santacruz Biotech, CA) was specific to a peptide mapping near the N-terminus of HSV1-TK.

### Flow cytometry

Dendritic cells, cells from DC-lymphocyte co-culture experiment, or splenic lymphocytes, thymocytes or lymph node cells harvested from vaccinated mice were washed twice with Dulbecco's phosphate-buffered saline (D-PBS) and suspended to a concentration of 1 × 10^6 ^cells in 100 μl D-PBS containing 1% FBS for staining with appropriate fluorochrome-conjugated antibodies (Table 2). After 30 min of incubation with antibodies, the cells were washed. The cells stained with biotin-conjugated antibodies were further incubated with streptavidin-APC or streptavidin-PE-texas red conjugate for 20 min. Depending on the number of fluorochromes, the cells were assayed either with a FACS Calibur or Influx flow cytometer (BD Biosciences, CA). The appropriate isotype-matched control antibodies were used to determine the levels of background staining. The histogram and dot plot data were collected and analyzed using either Summit v 4.3 (Dako Colorado Inc, CO), or FlowJo software (Flow Jo, OR) programs. We gated the positive cells in the histogram charts and obtained mean fluorescent intensity (MFI) values.

**Table 2:** List of fluorochrome-conjugated antibodies.

Cells	Fluorochrome-conjugated antibodies for staining DC or lymphocytes
DCs	Fluorescein isothiocyanate-conjugated (FITC)-CD14, CD3, CD86, CD62L; phycoerythrin (PE)-CD11c, CD45R, CD80, PDCA-1; Biotin or allophycocyanin (APC)-CD40, MHC class II (I-A/I-E), 120G8 and B220.

Cells from DC-lymphocyte co-culture	FITC-CD69, PE- CD8b (Ly-3), APC-CD4, PerCP-Cy5.5-CD25.

Splenic lymphocytes, thymocytes and lymph node cells	FITC- CD69, PE-CD127, Pacific orange-CD4, APC-CD8, PerCpCy5.5-CD25, Pacific blue-CD44, APC-Cy7-CD62L, Biotin-CCR7

### DC-lymphocyte co-culture experiment

For the DC-lymphocytes co-culture experiments, the DC to splenic lymphocytes ratios and time of incubation were optimized in the pilot experiments. Finally, in the comprehensive experiments, the nontransfected and transfected DCs (2dDC and 4dDC) were co-cultured with splenic lymphocytes (1 DC: 64 T cells) for a period of 24 h. The activation of T cells was determined on the basis of staining of cells with CD4, CD8 (T cell markers), CD25 and CD69 (activation markers)-specific antibodies.

### Cytokine analysis in cell-free supernatants of DCs

The cell-free supernatants collected from pVR1012-Ag2/PRA-cDNA-transfected and non transfected cells were analyzed for the secreted cytokines. The amounts of cytokines: tumor necrosis factor (TNF)-α, interleukin (IL)-6, IL-10, IL-12p70 and interferon (IFN)-γ, were measured in cell-free supernatants using mouse inflammation cytometric bead array kit (BD Biosciences, CA). Briefly, 50 μl of 1:10 and 1:100 diluted cell-free supernatants and diluted cytokine standard solutions (20-2500 pg/ml) were mixed with 50 μl of cytokine capture antibody mix and 50 μl of PE detection reagent. The reaction mixtures were incubated for 2 h, washed and resuspended in wash buffer. The samples were then assayed on FACS Calibur flow cytometer. The acquired data were analyzed for the amounts of cytokines in samples with the BD Cell Quest and CBA Array software programs.

### Monitoring of primary murine DC-based vaccine by PET-CT imaging

In order to study the *in vivo *trafficking of primary DCs, the cells were co-transfected with pVR1012-Ag2/PRA-cDNA and pVR1012-TK. The transfection protocol remained identical to that described above. The molecular imaging technique was based on the use of ^18^F-labeled 2'-fluoro-2'-deoxy-1ß-D-arabinofuranosyl-5-iodouracil (FIAU), a specific substrate for HSV1-TK. The cells expressing HSV1-TK phosphorylate ^18^F-FIAU to phospho-^18^F-FIAU, which is then incorporated, thus trapped, in the DNA and detected by PET system. Since untransformed mammalian cells do not express TK enzyme, normal mammalian cells do not phosphorylate FIAU, and therefore, are unable to entrap the probe and generate PET signal.

### Radiofluorination to synthesize ^18^F-FIAU

**Figure 13:**
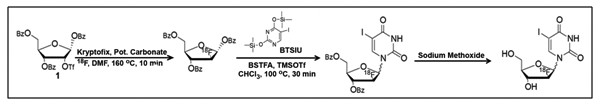
**A detailed schematic of ^18^F-FIAU synthesis for molecular imaging of DC distribution by PET**.

The PET imaging probe, ^18^F-FIAU was prepared as described earlier [21-23]. The fluorine-18 (^18^F) was produced by the (p, n) nuclear reaction on oxygen-18 (O-18)-enriched water with 11 MeV protons in an RDS-112 cyclotron (Midwest Medical Isotopes, Oklahoma City). The FIAU precursor (**1**) was prepared using following synthetic steps [22]. The synthetic scheme for generating ^18^F-FIAU as well as the precursor chemicals is shown in Figure 13 and described below.

#### 2-Deoxy-2-[^18^F]-fluoro-1,3,5-tri-*O*-benzoyl-α-D-arabinofuranose (2)

In a reaction vial containing 500 μl of Kryptofix solution (12 mg/ml), about 15 μl of K_2_CO_3 _(100 mg/ml) was added. The ^18^F-fluoride radioactivity was added to the reaction vial and the mixture was evaporated to dryness at 160°C under nitrogen gas. Azeotropic evaporation was performed with acetonitrile to remove all traces of water. The chemical precursor, 2-*O*-[(trifluoromethyl) sulfonyl]-1,3,5-tri-*O*-benzoyl-α-D-ribofuranose (**1**) in dimethylformamide (10 mg in 250 μl) was added to the reaction vial. The reaction mixture was heated at 160°C for 8 min. The reaction vial was transferred to a heated block (110°C), and the solvent was evaporated to about 50 μl under nitrogen. The vial was cooled to room temperature and dichloromethane (DCM, 5 ml) was added to the vial. The solution was then passed through a Sep-Pak silica cartridge (ChromTech, IL) to remove unreacted ^18^F-fluoride. The ^18^F-labeled compound **2 **was obtained in the DCM eluate with 50% radiochemical yield (RCY).

#### 1-(2'-Deoxy-2'-[^18^F]-fluoro-3,5-di-*O*-benzoyl-D-arabinofuranosyl) pyrimidines (3)

The DCM solution of compound **2 **was dried under nitrogen, and the residue was re-dissolved in chloroform (200 μl). To the solution of **2**, 2,4-bis-O-(trimethylsilyl)-5-iodouracil (200 μl of 0.252 M solution in acetonitrile), trimethyltrifluromethane sulfonate (20 μl) and bis-(trimethylsilyl)trifluoro acetamide (20 μl) were added. The reaction mixture was heated at 100°C for 30 min, and passed through Sep-Pak silica cartridge to obtain compound **(3) **as isomeric mixture in chloroform.

#### 1-(2'-Deoxy-2'-[^18^F]-fluoro-D-arabinofuranosyl) pyrimidines (4)

Compound **3 **was dried and the residue was added with acetonitrile (500 μl) and 250 μl of sodium methoxide (0.5 N) solution The mixture was heated at 100°C for 10 min, and the alkalinity was neutralized by adding 1.0 ml of 0.1% acetic acid. The de-protected ^18^F-FIAU was obtained by passing the mixture through a Sep-Pak C-18 column to collect the ^18^F-FIAU as a combination of α and β isomers. The isomeric mixture was concentrated and injected into an HPLC column (Phenomenex Luna column, 5 μm, 250 × 4.6 mm). Pure β-isomer of ^18^F-FIAU was collected as a peak with retention time of 9.8 min using a gradient system (5% to 100% acetonitrile in water over 25 min) at 254 nm wavelength. The product (Compound **4**) was dried and dissolved in saline with an overall RCY of 30-35%, and >95% radiochemical purity. The purity of the product was confirmed by injecting a small fraction of the finished product into an analytical HPLC, and spiking it with authentic FIAU (Moravek Biochemicals, Brea, CA).

### Measurement of HSV1-TK enzyme activity in transfected cells

Prior to *in vivo *imaging, we measured the TK enzyme activity in pVR1012-TK-transfected DCs using following methods.

#### HSV1-TK enzyme activity assay

The HSV1-TK enzyme activity was determined in pVR1012-TK transfected JAWS II DCs using ^18^F-FIAU as well as standard ^3^H-pencyclovir as substrates [49]. After 48 h of transfection, the transfected cells were lysed in a buffer containing 10 mM Tris-HCl, 3 mM of β-mercaptoethanol (Invitrogen-Gibco, CA), 0.5% Igepal CA-630, 25 mM sodium fluoride (Strem Chemicals, MA). The total protein in the cell lysate was analysed using bicinchonic acid protein assay kit (Pierce, IL). About 50 μg of total cell lysate protein was added to the TK reaction buffer (0.24 M NaH_2_PO_4_. H_2_O, pH 6.0 and 0.024 M ATP, pH 7.0) containing 150 μCi of ^18^F-FIAU, or 20 μCi of ^3^H-pencyclovir. The enzymatic reaction was allowed to occur at 37°C for 20 min. The reaction was stopped by adding ice-cold water. An aliquot (40 μl) of this reaction mixture was then blotted on Whatman DE81 filters (VWR, PA) and allowed to settle for 1 min. The filters were washed with a solution containing 4 mM of ammonium formate and 10 μM of thymidine in 95% ethanol. Finally, the dried-filters were placed in vials with (^3^H) or without (^18^F) scintillation fluid and radioactive counts (counts per min; cpm) were read on a Cobra II Autogamma counter (Packard Biosciences, CT) or Beckman Coulter LS6500 (Beckman Coulter, CA). Percent probe phosphorylated was calculated using the following equation:

$$\begin{array}{l} \text{Percent probe phosphorylated per }\mu \text{g per min}\\ \;\;=\frac{\left(\text{Total cpm}-\text{Background cpm}\right)}{\left(\text{Protein }\mu \text{g}\times \text{Reaction Time}\times \text{cpm in }3\mu \text{l HSV}1\text{-TK Reaction Buffer}\right)}\times 100 \end{array}$$

#### Uptake of ^18^F-FIAU by pVR1012-TK-transfected DCs [49-51]

After 48 h in transfection, the cells were washed with Dulbecco's PBS and incubated with 25 μCi ^18^F-FIAU in α- MEM medium without FBS at 37°C in 5% CO_2 _incubator. After 4 h incubation, cell-free medium was collected. The cells were washed with PBS and harvested in 100 μl of 0.1 M NaOH. The cell-associated and medium supernatant-associated radioactivity (cpm) was counted in Cobra II Autogamma gamma counter (Packard Biosciences, Meridian, CT). Percent uptake was normalized with the total number of cells in each well and calculated using the following formula:

$$\text{Percent Uptake}=\frac{\left(\text{Cell-associated cpm}\right)}{\left(\text{Cell-associated cpm}+\text{Medium-associated cpm}\right)}\times 100$$

### Intranasal immunization of syngeneic mice with primary DCs

The cells were transfected with pVR1012-Ag2/PRA-cDNA and pVR1012-TK plasmid DNAs, as described above. After 24 h of transfection, the syngeneic BALB/c mice were immunized with transfected DCs (1-1.5 × 10^6 ^DCs suspended in sterile, low-endotoxin 30-40 μl PBS per mouse) via intranasal route [23]. The control mice received DCs transfected with a vector plasmid DNA.

### PET-CT imaging

The PET-CT imaging was performed on vaccinated mice on days 2 and 7 of immunization. Radiolabeled ^18^F-FIAU was intravenously injected in mice via tail vein. The probe was allowed to distribute for 2 h before PET-CT imaging was performed. Mice were anesthetized with 2% isoflurane in an oxygen stream and positioned in the field of view (FOV) of X-PET/X-O-CT machine (Gamma Medica-Ideas, Northridge, CA). A fly-mode CT was acquired (2 min) to establish anatomical landmarks before re-positioning the animal for PET imaging. About 20 min list-mode PET data was acquired. The acquired image data were reconstructed using filtered back projection algorithm. Both PET and CT images were fused together using Amira 3.1 software provided with the imaging system.

### Biodistribution of ^18^F radioactivity

Mice were euthanized 140 min after injecting ^18^F-FIAU, and various organs and/or tissues (heart, lung, liver, spleen, stomach, intestine, kidney, blood, etc.) were excised under aseptic conditions. The organs were weighed, and the associated ^18^F radioactivity was counted in an automated gamma counter (Cobra II, Perkin-Elmer). The organ-associated counts were expressed as percent of injected dose per gram of tissue.

### T cell phenotype and Ag2/PRA-specific response in DC-vaccinated mice

In a separate set of DC-vaccinated mice, the lung, thymus, spleen and lymph nodes (superficial cervical, axillary, brachial and inguinal) were harvested on day 7 and lymphocytes were isolated using Lympholyte M density gradient solution as described above.

The splenic lymphocytes, thymocytes and lymph node cells were stained with antibodies conjugated to 8 different fluorochromes (Table 2). Single fluorochrome-conjugated stained lymphocytes were used for appropriate gating and to confirm no spill over. The T cell phenotype was then studied by flow cytometry. The percent number of CD4+ and CD8+ central memory (T_CM_) and effector memory (T_EM_) populations were noted on the basis of expression of CD62L and CCR7.

The T cell response against Ag2/PRA protein was assessed using ELISPOT assay as per the manufacturer's instructions (eBioscience, CA). Briefly, the wells of PVDF membrane ELISPOT assay plates (Millipore, CA) were coated with capture antibody to IFN-γ, IL-4 or IL-17A. The cells were seeded onto the antibody-coated wells at the density of 0.8-32 × 10^6 ^cells/ml and incubated in RPMI medium containing 10% FBS and antibiotics for 48 h in presence of recombinant Ag2/PRA (1 μg/ml). Subsequently, the biotinylated detection antibody, streptavidin-horse-radish-peroxidase conjugate and substrate solution were added, and development of spots was monitored. The wells were washed and air-dried. The spots were counted using a dissecting microscope. The frequency of cytokine-secreting, antigen-specific cells on the filtration membranes was calculated as the number of spots in the presence of Ag2/PRA minus the number of spots per equal number of cells in medium alone. Finally, the numbers of cytokine secreting cells harvested from mice immunized with vector plasmid DNA transfected DCs were subtracted from those in DC-vaccinated mice.

### Statistics

The results were analyzed by Student t-test for statistical significance using Prism software (Graphpad, San Diego, CA). The significant difference between the experimental and control groups was noted at p < 0.05.

## Authors' contributions

**PV **conducted the experiments under the guidance of **SA **and helped with data analysis. **PL **synthesized PET-imaging probe: FIAU. **VDA **performed imaging experiments, analyzed the biodistribution data and contributed in manuscript compilation. **CK **provided technical support and performed flow cytometric staining and ELISPOT assays. **NS **helped in cloning of the plasmid constructs. **SA **conceived and designed the study, prepared vaccine, conducted experiments, performed statistical analysis and wrote the manuscript. All authors read and approved the final manuscript.
